# Virus infection pattern imprinted and diversified the differentiation of T-cell memory in transcription and function

**DOI:** 10.3389/fimmu.2023.1334597

**Published:** 2024-01-09

**Authors:** Yuan Wang, Xinyue Mei, Zhengfang Lin, Xiaoyun Yang, Jinpeng Cao, Jiaying Zhong, Junxiang Wang, Li Cheng, Zhongfang Wang

**Affiliations:** ^1^ State Key Laboratory of Respiratory Disease & National Clinical Research Center for Respiratory Disease, Guangzhou Institute of Respiratory Health, the First Affiliated Hospital of Guangzhou Medical University, Guangzhou Medical University, Guangzhou, Guangdong, China; ^2^ Guangzhou National Laboratory, Bioland, Guangzhou, Guangdong, China

**Keywords:** CMV, EBV - Epstein-Barr virus, SARS-C0V-2, memory T cell, T cell differentiation

## Abstract

**Introduction:**

Memory T (Tm) cells are a subpopulation of immune cells with great heterogeneity. Part of this diversity came from T cells that were primed with different viruses. Understanding the differences among different viral-specific Tms will help develop new therapeutic strategies for viral infections.

**Methods:**

In this study, we compared the transcriptome of Tm cells that primed with CMV, EBV and SARS-CoV-2 with single-cell sequencing and studied the similarities and differences in terms of subpopulation composition, activation, metabolism and transcriptional regulation.

**Results:**

We found that CMV is marked by plentiful cytotoxic Temra cells, while EBV is more abundant in functional Tem cells. More importantly, we found that CD28 and CTLA4 can be used as continuous indicators to interrogate the antiviral ability of T cells. Furthermore, we proposed that REL is a main regulatory factor for CMV-specific T cells producing cytokines and plays an antiviral role.

**Discussion:**

Our data gives deep insight into molecular characteristics of Tm subsets from different viral infection, which is important to understand T cell immunization. Furthermore, our results provide basic background knowledges for T cell based vaccine development in future.

## Introduction

A remarkable feature of adaptive immunity is “immunological memory” (1), which is established during primary antigen encounters and can be persistent without residual antigens. The “memory” confers on the host a rapid, vigorous immune response to reinfection of given pathogens and provides long-term protection (2). Memory T cells are an important component of immunological memory that is highly specific and diverse. It is well known that memory T cells consist of many subpopulations that are highly heterogeneous (3, 4), but how these heterogeneous subpopulations of memory T cells are defined and how they differentiate into different phenotypes and diverse functions are not well known.

Very few phenotype markers have been discovered to define the differentiation and heterogenicity of memory T cells. Lanzavecchia first defined “central memory” T cells and “effector memory” T cells by chemokine receptor CCR7 expression (5), although people found that it is not always appropriate in all cases. On the other hand, the location of residential memory T cells from different tissues will influence the phenotype and function (6–9). For example, memory T cells located in bone marrow have a higher proliferation rate than those in lung tissue (10–12). Furthermore, the antigen dose, antigen exposure time, and duration of acute or persistent inflammation will also influence the differentiation, phenotype, and function of memory T cells. This heterogeneity of the memory T-cell repertoire is more pronounced when it is primed with different viruses. The extent and frequency of primed antigen determine the phenotype and function of virus-specific memory T cells (13, 14). For instance, CMV and EBV are viruses that both belong to Herpesviridae and are classified as chronic persistent infection viruses. However, CMV-specific Tm is remarkable for its particular CD8+ T-cell inflation (15), whereas EBV-specific T cells typically contract with the decrease in viral load. Meanwhile, SARS-CoV-2 can cause acute infection and may induce another T-cell memory differentiation pattern. Previous definitions of memory T cells are more derived from acute influenza infection and chronic viral infection, such as LCMV. However, it is not yet clear what the heterogeneity between the same cell subpopulations induced by different viruses is. What markers can be used to clearly describe the differentiation landscape of memory T cells primed in different viral infections is also not clear.

In this study, we used CMV-, EBV-, and SARS-CoV-2-specific Tm cells as examples to investigate the differences among Tm cells primed with different viral infections. A comprehensive comparison was made on these Tms, from subpopulation composition, cell activation, and metabolism to specific gene expression and transcriptional regulation. We found that CMV and EBV are different in subpopulation composition and that the same subpopulation of Tm cells has a viral-specific expression pattern. All these basic differences contribute to the functional diversity of different viral-specific Tms.

## Materials and methods

### Sample declaration and treatment

Blood samples used for EBV single-cell data analysis were obtained from one healthy adult donor with plentiful IFNg^+^ T cells after EBV peptide pool stimulation. It was approved by the Guangzhou Blood Center, China. The use of the blood samples was approved by the Health Commission of Guangdong Province and Guangzhou Institute of Respiratory Disease. Blood samples used in CMV and SARS-CoV-2 single-cell data analysis belong to a sample set of our previous study (16), which contained eight convalescents infected with CMV and SARS-CoV-2. Another five convalescents samples were also collected for further validation experiments. The convalescents in this study were defined as people that recovered (diagnosed with clinical nucleic acid testing and CT results) for more than 2 months. All information of samples used in this study is summarized in **Supplementary Table 1**. The study is approved by the Ethics Commission of the First Affiliated Hospital of Guangzhou Medical University (No. 2020-51). The signed consent forms from all the participants were obtained.

PBMCs were isolated by Ficoll-Paque (Cytiva) density gradient centrifugation. PBMCs from donors were stimulated with EBV, CMV, or SARS-CoV-2 peptides at 37°C for 16 h, with IL-2 but without GolgiPlug added. Cells that secreted IFN-γ were detected by an IFN-γ secretion assay kit (Miltenyi). Briefly, 1 × 10^6^ cells were resuspended in 90 µl of cold medium and 10 µl of IFN-γ catch reagent. After incubation for 5 min on ice, warm medium was added, and the cells were incubated at 37°C for 45 min. The cells were resuspended in 90 µl of cold buffer and 10 µl of IFN-γ detection antibody (APC) with other surface antibodies (Live–Dead V500, anti-human CD3-FITC (clone HIT3a), CD4-APC-H7 (clone RPA-T4), and CD8-PE-Cy7 (clone SK1)) for 20 min on ice before flow cytometry analysis. Subsequently, CD3^+^CD4^+^IFN-γ^+^ cells and CD3^+^CD8^+^IFN-γ^+^ cells were sorted by a FACSAria III flow cytometer.

### Peptide pool construction

The peptide pool construction was consistent with our previous published methods (15, 16). SARS-CoV-2-specific peptides were designed and synthesized against the following proteins: the entire antigen region of spike (S), nucleocapsid (N), membrane (M), and envelope (E). A total of 487 15-mer SARS-CoV-2 epitopes (overlapping by 11 amino acids) were generated with an online peptide generator (Peptide 2.0) and were synthesized by GL Biochem Corporation (Shanghai) with a purity of over 80%.

There were 383 15-mer (overlapping by 11 amino acids) CMV-specific peptides designed and synthesized to span the entire proteins of the HCMV pp65, IE-1, and IE-2. The EBV peptide pool contains 1,686 peptides that cover the EBV protein of BZLF1, BRLF1, BMRF1, BMLF1, EBNA1, EBNA2, EBNA3A, EBNA3B, EBNA3C, LMP1, LMP2A, and LMP2B. The length of each EBV-specific peptide was also 15-mer with 11 overlapping residues between neighboring peptides. The design and generation of these peptides were all with the online peptide generator (Peptide 2.0) and synthesized by GL Biochem Corporation (Shanghai) with a purity of >80%.

Each peptide was dissolved in DMSO with a concentration of 20 mM to form a stock. Full information of peptides used for EBV-, CMV-, and SARS-CoV-2-specific T-cell stimulation can be found in **Supplementary Table 2**.

### scRNA-seq library construction and sequencing

For EBV samples, single cells were encapsulated in droplets using 10× Genomics GemCode Technology and processed according to the manufacturer’s instructions. In brief, every single cell and every transcript were barcoded with a sample index and unique molecular identifier. Libraries were generated and sequenced from cDNAs using the Chromium Next GEM Single Cell 5′ Reagent Kits v1.1. The Single Cell 5′ Protocol was used to produce Illumina-ready sequencing libraries.

For CMV and SARS-CoV-2 samples, sample tag labeling was performed using a BD Human Single-Cell Multiplexing Kit (Cat. No. 633781) and processed on a BD Rhapsody™ Cartridge Reagent Kit (Cat. No. 633731) following the user’s manual. Single-cell mRNA, AbSeq barcodes, and SampleTag barcodes were all captured by BD Rhapsody beads coated with poly(T) oligonucleotide, with a unique cell barcode and molecular barcode on each bead. Single-cell cDNA synthesis and library amplification were performed following the manufacturer’s protocol by using a BD Rhapsody™ cDNA Kit (Cat. No. 633773) and BD Rhapsody™ WTA Amplification Kit (Cat. No. 633801). The cDNA library was constructed through two rounds of PCR for whole-transcriptome analysis and single-cell multiplexing analysis. Finally, eight cycles of PCR were performed for all elements following the manufacturer’s instructions. All PCR libraries were quantified using an Agilent Bioanalyzer 2100 and pooled. Pooled libraries were sequenced on NovaSeq with PE150.

### scRNA-seq data alignment and analysis

The scRNA-seq of EBV-stimulated T cells was aligned to reference GRCH38 and quantified with Cell Ranger software. The scRNA-seq of CMV- and SARS-CoV-2-stimulated T cells was also aligned to reference GRCH38 but with the BD Rhapsody WTA pipeline in version 1.9.1. Both data from 10x and BD were further analyzed with the Seurat R package. Cells with more than 25% mitochondrial RNA expression were filtered out. Additionally, cells with fewer than 500 RNA molecules or fewer than 250 expressed features were discarded. Because the ranges of the RNA molecule count and feature count were different between 10x and BD sequencing, we used 1.5-fold IQR as the threshold to remove cells with outlier values.

### Batch effect correction

To integrate the data sequenced with different platforms, in addition to SCT normalization, *Harmony* was also performed to correct the batch effect. As default, integration was performed with the top 3,000 highly variable genes. Furthermore, because the total read count is quite different between 10x and BD, the gene expression matrix was normalized with the iSMNN method (17), and the corrected matrix was used for further analysis.

### Dimensionality reduction and cluster annotation

Dimensionality reduction was performed with Harmony after SCT transformation, and cell clustering was carried out with 50 PCAs and with a resolution of 0.5. Then, UMAP was conducted on the Harmony result. To annotated the cell clusters, cell type-related markers were collected from different literatures (18–21) and R&D system websites (https://www.rndsystems.com/cn/resources/cell-markers/immune-cells/helper-t-cells). Th0 was defined as CD4^+^ T cells with CCR7 and SELL expression to keep consistency with the naming of other defined T helper cells, such as Th1 and Th17.

### Validation datasets

In case the results from a single donor of EBV is individual-specific, a validation dataset was collected from Zendo6952657. To keep the same status with our own sample, donor4 with a CMV- and EBV-negative status was selected as convalescent. In this dataset, viral-specific memory T cells were captured with the peptide-MHC method. The same data processing pipeline and cell-type gene markers were used as our own data in this validation dataset.

### Experiment validation

PBMCs from five convalescent (negative of NAT) donors were isolated from heparinized whole blood by density gradient sedimentation using Ficoll–Paque according to the manufacturer’s instructions (GE Healthcare, 17-1440-02). The PBMCs (5 × 105) were cultured in complete RPMI (c-RPMI, RPMI 1640 medium (Gibco)) enriched with supplements, including 10% heat-inactivated FBS (Biological Industries, Beit HaEmek, Israel), 100 μM MEM non-essential amino acids (Gibco), 100 U/mL penicillin (Gibco), 0.1 mg/mL streptomycin (Gibco), 2 mM L-glutamine (Gibco), 25 mM HEPES (Gibco), 55 μM 2-mercaptoethanol (Gibco), and 1 mM sodium pyruvate (Gibco).

PBMCs were stimulated with EBV peptide pools (1,623 peptides, 125 nM of each peptide), CMV peptide pools (383 peptides, 250 nM of each peptide), or SARS-CoV-2 peptide pools (481 peptides, 125 nM of each peptide) at 37°C for 12 h, with IL-2 but without GolgiPlug added. Then, anti-CCR7-APC (BioLegend, clone G043H7, Cat# 353214) was added to the culture along with GolgiPlug (BD Biosciences, San Diego, CA, USA) for a 4-h stain at 37°C. After 4 h, cells were washed in PBS supplemented with 2% FBS (FACS buffer) and incubated with surface staining for 30 min at room temperature with the following antibodies: anti-CD3-BUV395 (BD Bioscience, clone SK7, Cat# 564001), anti-CD4-BV786 (BioLegend, clone RPA-T4, Cat# 300554), anti-CD8-BV605 (BioLegend, clone RPA-T8, Cat# 301039), anti-CCR7-APC (BioLegend, clone G043H7, Cat# 353214), and anti-CD45RA-FITC (BioLegend, clone HI100, Cat# 304106). The cells were then washed twice and incubated with Live/Dead Aqua V510 for 15 min at room temperature. After fixation and permeabilization with Cytofix and Perm (BD Bioscience, Cat# 554714) on ice for 20 min, intracellular staining was performed on ice for 30 min with anti-TNF-PE-Cy7 (BD Bioscience, clone MAb11, Cat# 557647) and anti-IFNγ-PE (BD Bioscience, clone B27, Cat# 554701). After the final wash, the cells were resuspended in 200 μL FACS buffer. A FACSFortessa instrument (BD Bioscience) was used to acquire data, which were analyzed using FlowJo software (Treestar).

### Differential gene expression analysis and functional annotation

The differentially expressed genes were identified with the Seurat function “FindAllMarkers” or “FindMarkers”. The gene functional annotation for given gene sets was performed with the “clusterProfiler” R package.

### Transcription factor regulatory network inference

To identify the viral-specific active TF, “SCENIC” was carried out on our single-cell data with the default parameters. The motif information was obtained from “https://resources.aertslab.org/cistarget/databases/homo_sapiens/hg38/refseq_r80/mc9nr/gene_based/”. The correlation method was specified to “spearman.” Further network visualization of the inferred TF regulatory network was performed by “Cytoscape”.

### Correlation analysis

To identify the genes related to the switch between CD28 and CTLA4 expression in T cells, cells with both CD28 and CTLA4 expression were selected. The ratio of CD28 to CTLA4 in each cell was taken as an index to indicate cell activity. Then, Pearson correlation was performed between each gene in our expression matrix (except CD28 and CTLA4). All the positively correlated genes with FDR less than 0.05, which were calculated with the Benjamini and Hochberg method, were used to perform further gene functional annotation analysis.

### Pathway score

To quantify the activity of each cell in the NF-κB pathway, first, the genes in this pathway were acquired by the R package “KEGGREST” with KEGGID “hsa04064.” Then, the pathway score was calculated with the “AddModuleScore” function in Seurat. The plot was created by RidgePlot according to the added score.

## Results

### The same subsets of memory CD8+ T cells in acute/chronic viral infection differ greatly at the transcriptome level

**Figure 1:**
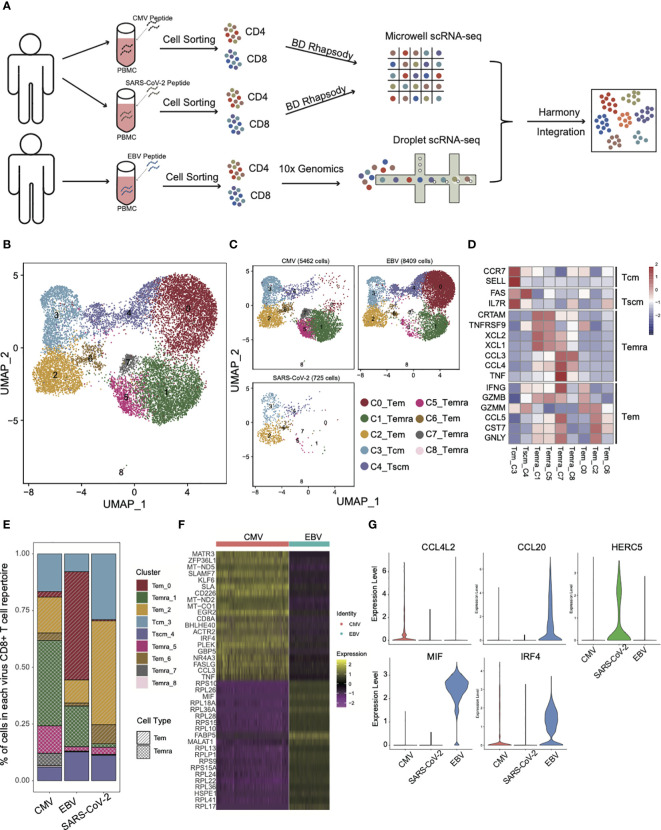
The landscape of CMV-, EBV-, and SARS-CoV-2-specific CD8+ T cells. **(A)** The pipeline for sample collection, treatment, and single-cell RNA-sequencing data preparation. **(B, C)** The UMAP figure of all CD8 cells from three different viruses; merged together in b and shown separately in c. The total CD8+ cell number for each of viruses was shown above each panel. The color denotes different cell clusters as the legend shows. **(D)** The mean expression of each gene in each cell cluster is presented in different colors, as shown in the legend color bar. The values were row scaled. The marker genes for different cell types were listed on the left. **(E)** The proportion of different cell clusters in the T-cell repertoire primed with different viruses is displayed in a stacked bar plot. The different colors represent different cell clusters the same as in figure **(B, C)**. The pattern of the bar indicates the cell types listed in the legend. **(F)** The top 20 DEGs between CMV and EBV Temra found by the “findMarker’ program are shown with the heatmap. Each row represents one gene, and each column represents one cell. The color is the expression value of a given gene in the given cell. **(G)** The top DEGs among CMV, EBV, and SARS-CoV-2 Tems. The expression for each gene in each cell is present with the violin plot. The red one is cells from CMV-primed, the green one from SARS-CoV-2 primed, and the blue one from EBV-primed.

To characterize the T-cell repertoires derived from different viral infections, CMV and EBV were used as chronic infection viral infection and SARS-CoV-2 was used as an acute infection. PBMCs were collected from convalescents that had ever been infected with EBV or CMV and SARS-CoV-2. Viral-specific CD4^+^ and CD8^+^ T cells were obtained by viral peptide stimulation followed by cell sorting (see Methods for details) (**Figure 1A**). Then, single-cell sequencing was performed with the 10x platform for EBV and BD Rhapsody for CMV and SARS-CoV-2. “Harmony” was used to integrate the data from two different platforms, and the performance was very satisfied (**Supplementary Figures 1A, B**). Virus-specific CD4^+^ and CD8^+^ T cells were separated based on the protein expression levels of CD4 and CD8.

As expected, different subsets of memory CD8^+^ T cells from three viral infections differed greatly in proportion (**Figures 1B, C**) and three effector T-cell subsets (cluster0,2,6, Tem), four terminally differentiated effector T-cell subsets (cluster1,5,7,8, Temra), one central memory T-cell subset (cluster3, Tcm), and one stem-like central memory T-cell subset (cluster4, Tscm) were defined according to expression of marker genes that were defined by previous studies (18–21) (**Figure 1D**). Temra, as a hallmark of CMV-specific T cells (22), accounted for the largest proportion (55.8%) of the CMV T-cell repertoire (**Figures 1C, E**). There is also a certain proportion of Temra in the EBV-specific T-cell repertoire (20%), although it is not the main group. This result is consistent with previous studies showing that the Temra phenotype is not the unique characteristic of CMV, and other chronic viruses can also elicit Temra with similar differentiation progression (22). The high frequency of Temra in CMV may be due to the “smoldering” character of CMV infection (23), which is reflected in the tiny CMV-specific CD8^+^ T-cell reactivation and antigen being rapidly extinguished. It made us to speculate that a high level of Temra in CMV may be caused by persistent stimulation, which can also lead to high expression of coinhibitory molecules that are associated with T-cell exhaustion (**Supplementary Figure 1C**). The difference in proportion of Temra and Tem between CMV and EBV was confirmed in an independent validation dataset. The same cell type criterion determination was performed as our own dataset (**Supplementary Figure 1E**) and the proportion of Temra in CMV was still dominated, whereas Tem is more in EBV (**Supplementary Figure 1D**). Additional experiment validation was also conducted in CMV and EBV, as well as SARS-CoV-2 specific T cells in another five convalescents donors. For all samples, the proportion of Temra in CMV was higher than that in EBV (**Supplementary Figure 1F**). These results indicate that although EBV and CMV are both laten viruses from Herpesviridae, the composition of T-cell repertoires are quite different.

Interestingly, in addition to the difference in the proportion of CD8 subsets, the same subset was also characterized by its own expression pattern in each virus. By taking all the Temra cells together, we found that CMV Temra cells highly expressed MATR3, ZFP36L1, MT-ND5, SLAMF7, and KLF6 than EBV (**Figure 1F**), within which SLAMF7 is documented to be upregulated in CMV-specific CD8^+^ T cells and downregulated in EBV-specific cells (24). EBV Temra is higher in FABP5, MIF, and MALAT1. These differentially expressed genes suggest that although Temra cells can be found in all three viral-specific T-cell repertoires, different viruses have their own characteristics on gene expression pattern.

Unlike CMV and EBV, SARS-CoV-2 has few Temra cells (**Figures 1C, E**). The main group of T cells was Tem in SARS-CoV-2 (54.8%) and EBV (59.2%). However, only 21.6% of CMV-specific CD8^+^ T cells were Tem cells. As can be noted in **Figure 1C**, the main Tem subgroups are different among CMV, EBV, and SARS-CoV-2. The major Tem subgroup is cluster 0 for EBV, and it is cluster 2 for CMV and SARS-CoV-2. By taking all the Tems (C0_Tem and C2_Tem and C6_Tem) together and comparing among them, we found that the Tems of CMV specifically overexpressed the chemokine CCL4L2, whereas those of EBV expressed CCL20 (**Figure 1G**). IRF4 is more active in both CMV and EBV than in SARS-CoV-2, which is consistent with a previous report that IRF4 can promote CD8^+^ T-cell exhaustion in chronic infection (25). HERC5 is demonstrated to be functional in SARS-CoV-2 infection and interferes with viral protein activity by modulating ISG15 signaling (26). Here, we found that HERC5 is highly expressed in SARS-CoV-2 Tem cells (**Figure 1G**). EBV Tem cells were peculiarly higher in MIF expression (**Figure 1G**), which has also been suggested by other studies for its function in EBV infection (27, 28).

**Figure 2:**
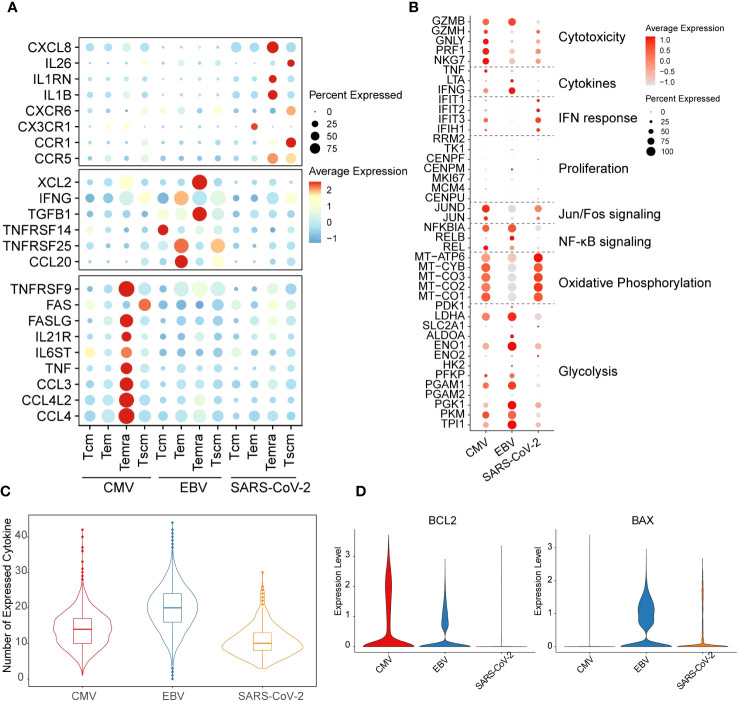
Distinct functions of different viral-specific T cells. **(A)** The cytokines with differential gene expression among different cell clusters. For each dot, the size represents the percentage of cells with non-zero expression of given genes (row) in given cell clusters (column). The color is the average expression as denoted in the legend. The virus-specific cytokines are listed on the left. **(B)** The expression for the marker genes of different functions is shown in the dot plot. The meaning for size and color is the same as that in figure **(A)**. **(C)** The number of cytokines with non-zero expression in each cell is plotted with the violin plot for each virus-specific T-cell repertoire. The quantile of the numbers is labeled with the boxplot. **(D)** The expression of gene BCL2 and BAX in each cell is plotted with the violin plot. The red violin indicates CMV-primed T cells, the blue one for EBV-primed cells, and the orange one for SARS-CoV-2-primed ones.

We found, for CMV, that the main cell type that expressed cytokine-related genes was Temra, which was enriched with CCL3/4, CCL4L2, TNF, IL6ST, IL21R, FASLG, and TNFRSF9 expression (**Figure 2A**). In contrast to CMV, Temra in EBV exclusively highly expressed XCL2 and TGFB1 whereas SARS-CoV-2 Temra highly expressed CXCL8, IL1RN, IL1B, and CCR5. Our results also showed that SARS-CoV-2 displayed extreme IL-26 and CCR1 gene expression activity in Tscm cells (**Figure 2A**). CCR1 was reported to be highly expressed in DCs in SARS-CoV-2 patients and is considered a potential therapeutic target clinically (29). Here, we provided further evidence implying that CCR1 is also highly expressed in SARS-CoV-2 Tscm cells. The distinction of functions for different T cells can, to a certain extent, be reflected on the expression of cytokine secretion-related genes.

Taken together, our results showed that CD8^+^ T cells derived from different viruses can form T-cell subgroups with specific transcription characteristics. However, whether different transcriptome patterns in the Tm of CMV, EBV, and SARS-CoV-2 indicate that they use different metabolism modes or have different functions is less defined.

### Metabolism and function are divergent for T cells from different viruses

It is generally considered that T cells undergo glycolysis during the “effector” phase, whereas naïve and memory T cells are inclined to undergo oxidative phosphorylation (30). Therefore, to further study the function and metabolism model of T cells from different viral infection patterns, function-related genes were collected from previous studies (31). In our results, EBV-specific CD8^+^ T cells were more prone to highly express glycolysis genes. In contrast, oxidative phosphorylation activation gene expression is much stronger in SARS-CoV-2. Unlike the other two viruses, CMV has gene expression on both metabolism pathways but with mild intensity compared with EBV and SARS-CoV-2 (**Figure 2B**). The cell metabolic state dynamically changes with T-cell function. SARS-CoV-2 and EBV are all enriched with the Tem subgroup; however, the high expression level of oxidative phosphorylation process in SARS-CoV-2 Tem led us to query whether they are still in the effector phase. Indeed, we found that cytotoxicity and general cytokine genes, such as IFNG and TNF, were all expressed at low levels in SARS-CoV-2 (**Figure 2B**). However, the expression of IFN response genes was highest in SARS-CoV-2 and lowest in EBV (**Figure 2B**). In addition, the genes in the NF-κB signaling pathway and Jun/Fos pathway are inactive in SARS-CoV-2, which further implies attenuation of effector function (**Figure 2B**). Additionally, the number of expressed cytokines was lowest in SARS-CoV-2 among the three viruses (**Figure 2C**). The reduction in cytokines can trigger the cell apoptosis process, and the downregulation of BCL2 and upregulation of BAX can also be seen in SARS-CoV-2 when compared with the other two viruses (**Figure 2D**). Therefore, all these results taken together, we proposed that the Tem in the SARS-CoV-2 stimulation model is likely undergoing a functional fading and memory phase in convalescents.

### CD4^+^ T cells are more concordant than CD8^+^ cells among different viral infections

**Figure 3:**
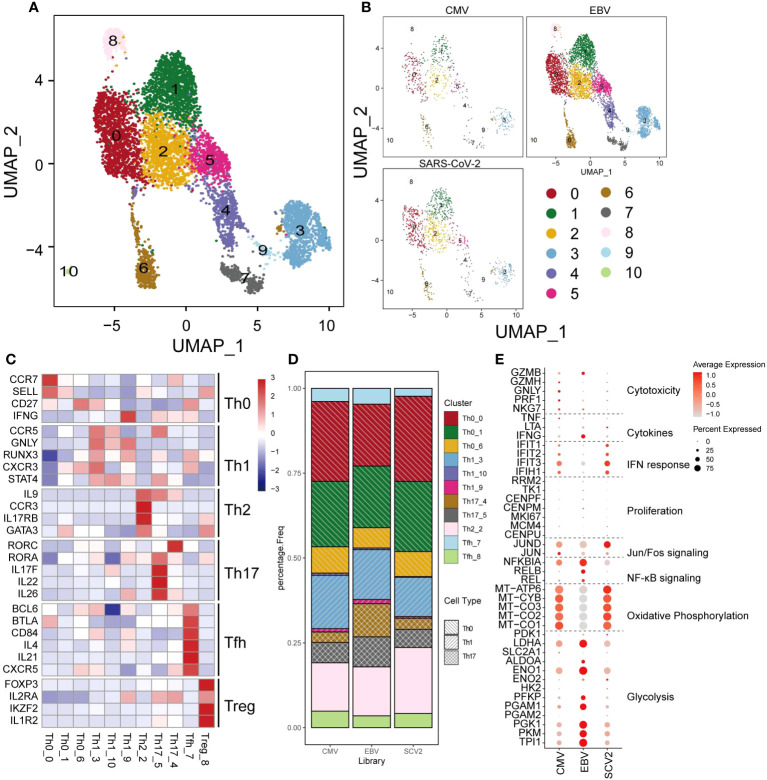
The landscape of CMV-, EBV-, and SARS-CoV-2-specific CD4+ T cells. **(A, B)** The UMAP figure of all CD4 cells from three different viruses, taken together in **(B)** and shown separately in **(C)**. The color denotes different cell clusters as the legend shows. **(C)** The mean expression of each gene in each cell cluster is presented in different colors as shown in the legend color bar. The values were row scaled. The marker genes for different cell types were separated with gap rows. **(D)** The proportion of different cell clusters in the T-cell repertoire primed with different viruses is displayed in a stacked barplot. The different color represents different cell clusters the same as in a-b. The pattern of the bar indicates the cell types listed in the legend. **(E)** The expression for the marker genes of different functions is shown in the dot plot. The size represents the percentage of cells with non-zero expression in given genes (row) and given cell clusters (column). The color is the average expression, as denoted in the legend.

CD4^+^ T cells are another crucial cell type in antiviral processes. However, unlike that in CD8^+^ T cells, the population of CD4^+^ T cells did not present much of a difference among the three viruses (**Figures 3A, B, D**). CD4^+^ T cells were classified as Th0, Th1, Th2, Th17, Tfh, or Treg cells based on marker genes (**Figure 3C**), and we only found some differences in Th17 cells, which showed a larger proportion than the other two viruses (18.6% in EBV, 9% and 8.6% in CMV and SARS-CoV-2) (**Figure 3D**). Cytotoxic and cytokine genes were hardly expressed in all CD4^+^ T cells (**Figure 3E**), which could be because the main cells responding to ex vivo peptide stimulation are CD8^+^ in CMV and EBV. Although SARS-CoV-2 CD4^+^ cells have a certain response to peptide stimulation, the production of cytokines was low in most studies. In line with CD8^+^ T cells, EBV CD4^+^ T cells show high expression in glycolysis genes, whereas CMV and SARS-CoV-2 are high in oxidative phosphorylation (**Figure 3E**). Most of the top differentially expressed genes between different viruses were related to metabolism, such as mitochondrial genes and ribosome genes (**Supplementary Figure 2**). In addition, we found that MIF and FABP5 were exclusively expressed in EBV. FABP5 is reported to be positively related to Th17 differentiation (32); therefore, we suggested that the high proportion of Th17 in EBV may be due to the high expression of FABP5.

### CD28 to CTLA4 transition regulates T-cell antiviral function

**Figure 4:**
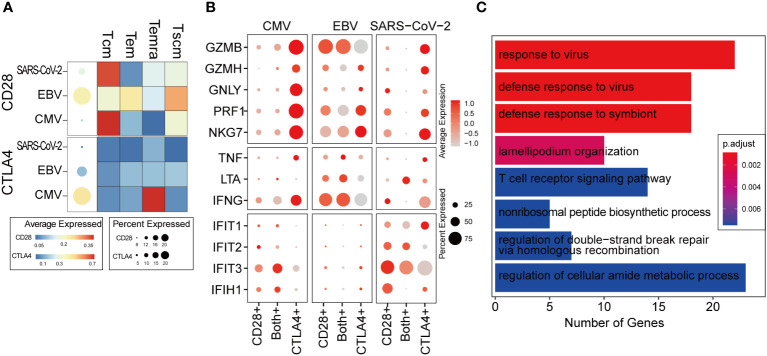
The ratio of CD28 to CTLA4 related with T-cell activity. **(A)** The average expression of CD28 and CTLA4 for all cells in given the virus-specific T-cell repertoire is shown in the dot plot. The size is the percentage of cells with non-zero value, and the color is the expression value as denoted in the legend color bars. The heatmap separates the T-cell repertoire into different cell types and with the color represents the average expression in given cell types for each virus. The value is row scaled. **(B)** Each dot indicates the average expression of a given gene (row) in a given cell cluster (column). The meaning of the dot size is similar with that in **(A)**. **(C)** The genes positively correlated with the CD28/CTLA-4 ratio was collected for functional enrichment analysis. The top most significantly enriched functions are listed in the barplot. The length of the bar represents the number of genes belonging to given functions. The color is an adjusted p-value.

CTLA4 transcription was immediately initiated when the T cell was activated. Intracellular CTLA4 accumulates on the surface of T cells by externalization and competes with CD28 binding to ligands and negatively regulates T-cell function (33). We studied the expression patterns of CD28 and CTLA4 at single-cell resolution to characterize the dynamic changes in T cells from activation to functional inhibition, which is also called “T-cell exhaustion.” In a total of 14,596 CD8^+^ T cells, only 29% expressed either CD28 or CTLA4 and 2.7% expressed both (**Supplementary Figure 3A**). CD28 was found to be hardly expressed in CMV when taking all the virus-specific CD8^+^ T cells into account, but a more detailed distinction shows that some CMV-specific Tcm cells still expressed CD28 (**Figure 4A**, upper panel). We found that regardless of CMV, EBV, or SARS-CoV-2 infection, cells with further differentiation potential presented relatively high CD28 expression, such as Tcm and Tscm. Temra shows the lowest expression of CD28 in CMV and EBV. In contrast, CTLA4 is extremely highly expressed in CMV Temra cells, which further indicates the mutually exclusive correlation between CD28 and CTLA4 due to the competitive binding of ligands (**Figure 4A**, lower panel).

We considered cells with only CD28 expression as T cells in the “activation phase,” cells with only CTLA4 expression as T cells in the “activity inhibition phase,” and cells with both gene expression as the “transformation phase”. Interestingly, the cytotoxic genes were highly expressed in the “inhibition phase,” which is inconsistent with our expected “function inhibition” (**Figure 4B**). However, it seems that the expression of cytotoxic genes, such as GZBM and IFNG, is commonly observed in exhausted T cells reported by other studies (34, 35). Most of the cells belonging to the “activation phase” in CMV and SARS-CoV-2 are Tcm (**Supplementary Figure 3B**), which is not the major cell type responsible for cytokine secretion (**Figure 2A**). Therefore, in CMV and SARS-CoV-2, cytokine and cytotoxic genes cannot be expressed as widely as in EBV (**Figure 4B**).

In the transformation phase, we hypothesize that the cells undergo a process in which the ratio of CD28/CTLA4 expression is gradually attenuated. To decipher the details of the alteration in molecular function during this process, a correlation analysis was carried out within cells in the transformation phase (see Methods for details). The genes that were positively correlated with the ratio of CD28/CTLA4 were found to be related to the “responding to virus” and “T cell receptor signaling pathway” (**Figure 4C**), which indicates that a higher CD28/CTLA4 ratio elicits a stronger T-cell response. For further validation, two public scRNA-seq datasets for PBMC samples from hospitalized COVID-19 patients were collected. The same pipeline and criteria were applied, and they all indicated the ratio of CD28/CTLA4 expression related to T-cell function (**Supplementary Figure 3C**).

### TF regulates viral-specific cytokine secretion and cell function

**Figure 5:**
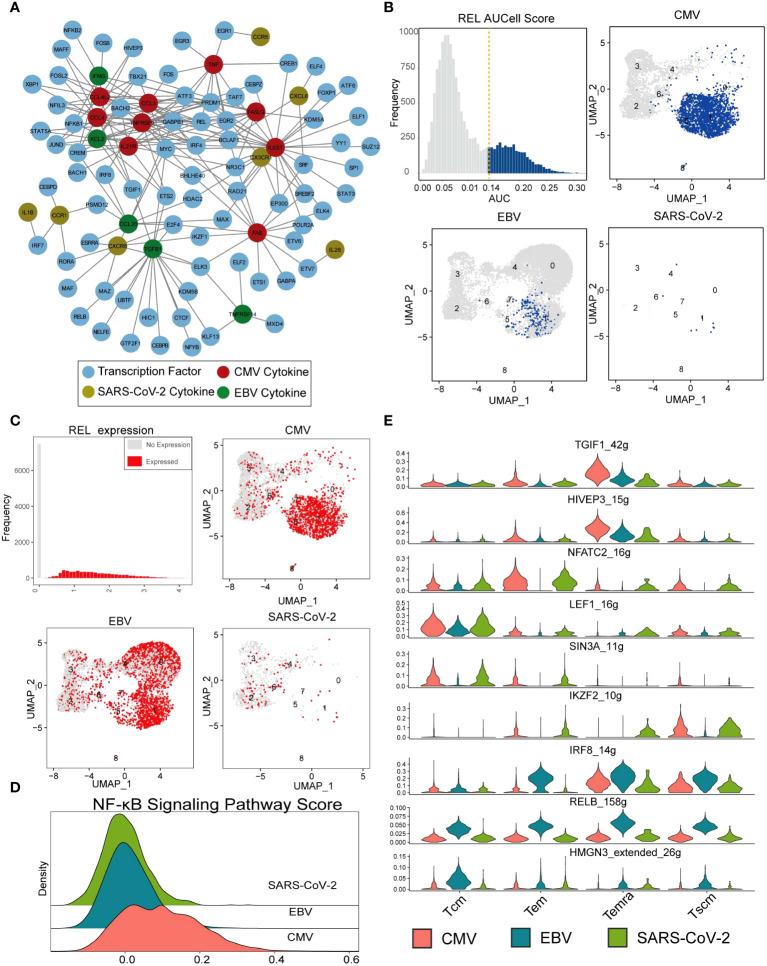
REL regulates cytokine expression in CMV-specific T cells. **(A)** The network constructed with cytokines in **Figure 2A** and their SCENIC imputed transcription factors. The nodes with different colors indicate CMV- (red), EBV- (green), and SARS-CoV-2-specific (gold) cytokines. Blue nodes are deduced TFs, and the lines represent the regulation relationship between cytokine and TF. **(B)** The top-left panel shows the REL AUC distribution, which is calculated with SCENIC. The x-axis is the AUC value, and the y-axis is the frequency of cells with a given AUC value. The dashed line labels the cutoff used to defined active regulon. The remaining three panels show the AUC value of each virus-specific T cell with UMAP plot. **(C)** Similar with that in figure **(B)**, with REL gene expression instead of AUC value. **(D)** The Ridge plot for the pathway score of the NF-κB signaling pathway. The a-axis is the pathway score, and the y-axis is the density of cells with a given score. **(E)** The violin plot shows the RSS score (regulon specificity score from SCENIC) (y-axis) for each TF in each virus-specific T-cell cluster (column).

It is generally accepted that transcription factors play a vital role in T-cell activation, reaction, and differentiation (36–38). In the first part of our results, we denoted that the expression of cytokines is viral specific. To examine the rationale behind this expression heterogeneity, a regulatory network inference analysis was performed with the SCENIC pipeline (39) on our single-cell data (see Methods for details). The analysis takes both gene coexpression and binding motifs into account to recognize the TF targets and takes “regulon” as the unit to estimate the activity of TF in each cell. According to the deduced regulatory results, the 23 viral-specific cytokines of interest (in **Figure 2A**) and their 76 TFs built up a network with a maximum cytokine degree of 28 for IL6ST and a maximum TF degree of 12 for GABPB1 (**Figure 5A**; **Supplementary Figures 4A–C**). In the CMV subnetwork (**Supplementary Figure 4A**), we found that eight out of nine CMV-specific cytokines were targeted by REL, which indicates its key function in CMV CD8^+^ Tm cells. REL is a subunit of the NF-κB family, and many studies suggest that the activation of CMV is related to the activity of REL (40, 41). In total, there were 3,100 CD8^+^ Tm cells denoted as “REL regulon” active in our data according to the AUCell score (with 0.14 as cutoff) (**Figure 5B**), and most of them were CMV-specific Temra cells (**Supplementary Figure 4D**), which is in line with cytokine expression in our previous results (**Figure 5B**). However, the expression of REL is not CMV specific. Tem in EBV also displayed high REL expression but little REL regulon activity (**Figure 5C**). This result further indicates that it is the ‘regulatory event’ rather than TF expression that is responsible for cytokine secretion. Furthermore, we also found that the activity of the NF-κB signaling pathway was higher in CMV than in EBV and SARS-CoV-2 (**Figure 5D**), which further suggests the potential roles of REL in both signal transduction and cytokine secretion regulation in CMV-specific Tm cells.

SCENIC identified 182 TF regulons with condition-specific activity (**Supplementary Figure 5**), such as the Tcm-specific TF “LEF1,” EBV-specific TF “IRF8,” “RELB,” and “HMGN3” and CMV/EBV Temra-specific TF “TGIF1” and “HIVEP3” (**Figure 5E**). All these TFs resulted in distinguished downstream gene expression and further distinctions in cell function for different viral infections.

## Discussion

It is generally agreed that the Tm repertoire primed by different viruses is divergent. However, it is still unclear how and to what extent they are different. Our results point out that in CMV it is mainly the Temra subpopulation that is dominant, whereas EBV and SARS-CoV-2 are the Tem subpopulations that take advantage. Our results also indicate that they are different in cytokine secretion, cell metabolism, and cell activation regulation. In our study, viral-specific Tm cells were activated by the viral peptide pool, which can capture all T cells primed by a given virus under current circumstances and are not limited by MHC restriction. However, the difference caused by different circumstances from different samples is inevitable. Further studies working in the same environmental system are still necessary.

The enrichment of Temra T cells is one of the characters of CMV (42). However, as another persistent virus, EBV comprises Tem rather than Temra cells. Our results suggest that the main functional cells in CMV are those with the Temra phenotype instead of the Tem phenotype. As our results indicated, SARS-CoV-2-specific Tm cells show high gene expression on oxidative processes. This result is reasonable because SARS-CoV-2 is an acute infectious virus, and most effector T cells enter the “memory phase” in convalescence. EBV is a persistent infectious virus, and most of the time, reactivation is asymptomatic, but EBV-specific Tm cells are active in the glycolysis process, which implies the “effector phase” is going through. CMV is quite different from EBV and SARS-CoV-2, not only in the phenotype of Tm but also in the metabolism process, which may be due to the accumulation of viral-specific T cells caused by inflation.

Costimulation is a secondary signal that is required for T-cell activation (43). In contrast, coinhibitors calm down the effective T cells and maintain the equilibrium of the immune system. Chronic and persistent infection results in a high level of coinhibitor expression; however, many studies have found that exhausted T cells expressed a high level of cytokines (34). We speculate that these results indicate that there is a “transition phase,” in which T cells have inadequate function and express both costimulators and coinhibitors. Indeed, our results demonstrate that T-cell activation decreases as the ratio of CD28/CTLA-4 expression decreases. This study provides a feasible prospect for antiviral therapy by retrieving costimulator expression in virus-specific T-cell populations in the future.

How cells transmit external stimuli into the nucleus and respond appropriately is another attractive field. By studying the transcription factor regulatory network, we found that the TF REL is more active in CMV than in EBV and SARS-CoV-2. In addition, as a key subunit of NF-κB, high activity of REL can be related with the increased signaling of the NF-κB pathway in CMV. Therefore, we suggest that CMV Tm cells transmit external stimuli through the NF-κB pathway and initiate the transcription of cytokines in the nucleus, such as CCL3, CCL4, and TNF. We also listed several possible TFs for EBV and SARS-CoV-2 Tms, such as IKZF2 in SARS-CoV-2 and IRF8 in EBV. In brief, our study provides comprehensive evidence that Tm primed by different viruses are quite different in phenotype, function, metabolism, transcription profile, and TF-mediated signal transduction.

## Data availability statement

The raw data and code were available according to requirements. The public data used for CD28 and CTLA4 transition phase correlation analysis validation were from GSE155223 (44) and GSE180578 (45). Only PBMC samples from COVID-19 patients were considered. 

## Ethics statement

The studies involving humans were approved by Health Commission of Guangdong Province and Guangzhou Institute of Respiratory Disease. The studies were conducted in accordance with the local legislation and institutional requirements. The participants provided their written informed consent to participate in this study.

## Author contributions

YW: Conceptualization, Formal analysis, Funding acquisition, Investigation, Visualization, Writing – original draft, Writing – review & editing. XM: Methodology, Validation, Writing – review & editing. ZL: Resources, Writing – review & editing. XY: Resources, Writing – review & editing. JC: Data curation, Writing – review & editing. JZ: Data curation, Writing – review & editing. JW: Data curation, Writing – review & editing. LC: Data curation, Writing – review & editing. ZW: Funding acquisition, Supervision, Writing – review & editing.

## References

[1] Xu A. Chapter2-basic knowledge of immunology. In: Amphioxus Immunity (2016) (Cambridge, MA, USA: Academic Press). p. 15–42.

[2] Welsh RM Selin LK Szomolanyi-Tsuda E. Immunological memory to viral infections. Annu Rev Immunol (2004) 22:711–43. doi: 10.1146/annurev.immunol.22.012703.104527 15032594

[3] Ahmadzadeh M Hussain SF Farber DL. Effector CD4 T cells are biochemically distinct from the memory subset: evidence for long-term persistence of effectors *in vivo* . J Immunol (1999) 163:3053–63. doi: 10.4049/jimmunol.163.6.3053 10477569

[4] Ahmadzadeh M Hussain SF Farber DL. Heterogeneity of the memory CD4 T cell response: persisting effectors and resting memory T cells. J Immunol (2001) 166:926–35. doi: 10.4049/jimmunol.166.2.926 11145669

[5] Sallusto F Lenig D Forster R Lipp M Lanzavecchia A. Pillars article: two subsets of memory T lymphocytes with distinct homing potentials and effector functions. Nature. 1999. 401: 708-712. J Immunol (2014) 192:840–4. 24443506

[6] Masopust D Vezys V Marzo AL Lefrancois L. Preferential localization of effector memory cells in nonlymphoid tissue. Science (2001) 291:2413–7. doi: 10.1126/science.1058867 11264538

[7] Bingaman AW Patke DS Mane VR Ahmadzadeh M Ndejembi M Bartlett ST. Novel phenotypes and migratory properties distinguish memory CD4 T cell subsets in lymphoid and lung tissue. Eur J Immunol (2005) 35:3173–86. doi: 10.1002/eji.200526004 16220537

[8] Masopust D Lefrancois L. CD8 T-cell memory: the other half of the story. Microbes Infect (2003) 5:221–6. doi: 10.1016/s1286-4579(03)00014-5 12681411

[9] Masopust D Vezys V Usherwood EJ Cauley LS Olson S Marzo AL. Activated primary and memory CD8 T cells migrate to nonlymphoid tissues regardless of site of activation or tissue of origin. J Immunol (2004) 172:4875–82. doi: 10.4049/jimmunol.172.8.4875 15067066

[10] Zhang X Dong H Lin W Voss S Hinkley L Westergren M. Human bone marrow: a reservoir for “enhanced effector memory” CD8+ T cells with potent recall function. J Immunol (2006) 177:6730–7. doi: 10.4049/jimmunol.177.10.6730 17082586

[11] Woodland DL Scott I. T cell memory in the lung airways. Proc Am Thorac Soc (2005) 2:126–31. doi: 10.1513/pats.200501-003AW PMC2713315 16113480

[12] Ely KH Cookenham T Roberts AD Woodland DL. Memory T cell populations in the lung airways are maintained by continual recruitment. J Immunol (2006) 176:537–43. doi: 10.4049/jimmunol.176.1.537 16365448

[13] Lefrancois L Marzo AL. The descent of memory T-cell subsets. Nat Rev Immunol (2006) 6:618–23. doi: 10.1038/nri1866 16868553

[14] Moulton VR Farber DL. Committed to memory: lineage choices for activated T cells. Trends Immunol (2006) 27:261–7. doi: 10.1016/j.it.2006.04.006 16684621

[15] Zhang J Cao J Zheng R Yu M Lin Z Wang C. The establishment of a cytomegalovirus -specific CD8(+) T-cell threshold by kinetic modeling for the prediction of post-hemopoietic stem cell transplant reactivation. iScience (2022) 25:105340. doi: 10.1016/j.isci.2022.105340 36325063 PMC9618782

[16] Wang Z Yang X Zhong J Zhou Y Tang Z Zhou H. Exposure to SARS-CoV-2 generates T-cell memory in the absence of a detectable viral infection. Nat Commun (2021) 12:1724. doi: 10.1038/s41467-021-22036-z 33741972 PMC7979809

[17] Yang Y Li G Xie Y Wang L Lagler TM Yang Y. iSMNN: batch effect correction for single-cell RNA-seq data via iterative supervised mutual nearest neighbor refinement. Brief Bioinform (2021) 22(5):bbab122. doi: 10.1093/bib/bbab122 33839756 PMC8579191

[18] Tian Y Babor M Lane J Seumois G Liang S Goonawardhana NDS. Dengue-specific CD8+ T cell subsets display specialized transcriptomic and TCR profiles. J Clin Invest (2019) 129:1727–41. doi: 10.1172/JCI123726 PMC6436856 30882366

[19] Szabo PA Levitin HM Miron M Snyder ME Senda T Yuan J. Single-cell transcriptomics of human T cells reveals tissue and activation signatures in health and disease. Nat Commun (2019) 10:4706. doi: 10.1038/s41467-019-12464-3 31624246 PMC6797728

[20] Milner JJ Nguyen H Omilusik K Reina-Campos M Tsai M Toma C. Delineation of a molecularly distinct terminally differentiated memory CD8 T cell population. Proc Natl Acad Sci USA (2020) 117:25667–78. doi: 10.1073/pnas.2008571117 PMC7568335 32978300

[21] Hong H Gu Y Sheng SY Lu CG Zou JY Wu CY. The distribution of human stem cell-like memory T cell in lung cancer. J Immunother (2016) 39:233–40. doi: 10.1097/CJI.0000000000000128 PMC4902324 27244531

[22] van den Berg SPH Pardieck IN Lanfermeijer J Sauce D Klenerman P van Baarle D. The hallmarks of CMV-specific CD8 T-cell differentiation. Med Microbiol Immunol (2019) 208:365–73. doi: 10.1007/s00430-019-00608-7 PMC6647465 30989333

[23] Snyder CM Cho KS Bonnett EL Allan JE Hill AB. Sustained CD8+ T cell memory inflation after infection with a single-cycle cytomegalovirus. PloS Pathog (2011) 7:e1002295. doi: 10.1371/journal.ppat.1002295 21998590 PMC3188546

[24] Loyal L Warth S Jurchott K Molder F Nikolaou C Babel N. SLAMF7 and IL-6R define distinct cytotoxic versus helper memory CD8(+) T cells. Nat Commun (2020) 11:6357. doi: 10.1038/s41467-020-19002-6 33311473 PMC7733515

[25] Man K Gabriel SS Liao Y Gloury R Preston S Henstridge DC. Transcription factor IRF4 promotes CD8(+) T cell exhaustion and limits the development of memory-like T cells during chronic infection. Immunity (2017) 47:1129–1141 e1125. doi: 10.1016/j.immuni.2017.11.021 29246443

[26] Mathieu NA Paparisto E Barr SD Spratt DE. HERC5 and the ISGylation pathway: critical modulators of the antiviral immune response. Viruses (2021) 13(6):1102. doi: 10.3390/v13061102 34207696 PMC8228270

[27] Wilson GL Young BG. Production of migration inhibitory factor (MIF) by human leukocytes following exposure to Epstein-Barr virus. Cell Immunol (1978) 38:147–56. doi: 10.1016/0008-8749(78)90040-0 208782

[28] Feng G Xu Y Ma N Midorikawa K Oikawa S Kobayashi H. Influence of Epstein-Barr virus and human papillomavirus infection on macrophage migration inhibitory factor and macrophage polarization in nasopharyngeal carcinoma. BMC Cancer (2021) 21:929. doi: 10.1186/s12885-021-08675-x 34407796 PMC8371777

[29] Law HK Cheung CY Sia SF Chan YO Peiris JS Lau YL. Toll-like receptors, chemokine receptors and death receptor ligands responses in SARS coronavirus infected human monocyte derived dendritic cells. BMC Immunol (2009) 10:35. doi: 10.1186/1471-2172-10-35 19505311 PMC2700820

[30] Michalek RD Rathmell JC. The metabolic life and times of a T-cell. Immunol Rev (2010) 236:190–202. doi: 10.1111/j.1600-065X.2010.00911.x 20636818 PMC2983473

[31] Adamo S Michler J Zurbuchen Y Cervia C Taeschler P Raeber ME. Signature of long-lived memory CD8(+) T cells in acute SARS-CoV-2 infection. Nature (2022) 602:148–55. doi: 10.1038/s41586-021-04280-x PMC8810382 34875673

[32] Li B Reynolds JM Stout RD Bernlohr DA Suttles J. Regulation of Th17 differentiation by epidermal fatty acid-binding protein. J Immunol (2009) 182:7625–33. doi: 10.4049/jimmunol.0804192 PMC2707838 19494286

[33] Rudd CE Taylor A Schneider H. CD28 and CTLA-4 coreceptor expression and signal transduction. Immunol Rev (2009) 229:12–26. doi: 10.1111/j.1600-065X.2009.00770.x 19426212 PMC4186963

[34] Miller BC Sen DR Abosy Al R Bi K Virkud YV LaFleur MW. Subsets of exhausted CD8(+) T cells differentially mediate tumor control and respond to checkpoint blockade. Nat Immunol (2019) 20:326–36. doi: 10.1038/s41590-019-0312-6 PMC6673650 30778252

[35] Beltra JC Manne S Abdel-Hakeem MS Kurachi M Giles JR Chen Z. Developmental relationships of four exhausted CD8(+) T cell subsets reveals underlying transcriptional and epigenetic landscape control mechanisms. Immunity (2020) 52:825–841 e828. doi: 10.1016/j.immuni.2020.04.014 32396847 PMC8360766

[36] Chen Y Zander R Khatun A Schauder DM Cui W. Transcriptional and epigenetic regulation of effector and memory CD8 T cell differentiation. Front Immunol (2018) 9:2826. doi: 10.3389/fimmu.2018.02826 30581433 PMC6292868

[37] Serfling E Berberich-Siebelt F Chuvpilo S Jankevics E Klein-Hessling S Twardzik T. The role of NF-AT transcription factors in T cell activation and differentiation. Biochim Biophys Acta (2000) 1498:1–18. doi: 10.1016/s0167-4889(00)00082-3 11042346

[38] Yu B Zhang K Milner JJ Toma C Chen R Scott-Browne JP. Epigenetic landscapes reveal transcription factors that regulate CD8(+) T cell differentiation. Nat Immunol (2017) 18:573–82. doi: 10.1038/ni.3706 PMC5395420 28288100

[39] Aibar S Gonzalez-Blas CB Moerman T Huynh-Thu VA Imrichova H Hulselmans G. SCENIC: single-cell regulatory network inference and clustering. Nat Methods (2017) 14:1083–6. doi: 10.1038/nmeth.4463 PMC5937676 28991892

[40] Johari YB Scarrott JM Pohle TH Liu P Mayer A Brown AJ. Engineering of the CMV promoter for controlled expression of recombinant genes in HEK293 cells. Biotechnol J (2022) 17:e2200062. doi: 10.1002/biot.202200062 35482470

[41] Bellas RE Lee JS Sonenshein GE. Expression of a constitutive NF-kappa B-like activity is essential for proliferation of cultured bovine vascular smooth muscle cells. J Clin Invest (1995) 96:2521–7. doi: 10.1172/JCI118313 PMC185913 7593644

[42] Derhovanessian E Maier AB Hahnel K Beck R de Craen AJM Slagboom EP. Infection with cytomegalovirus but not herpes simplex virus induces the accumulation of late-differentiated CD4+ and CD8+ T-cells in humans. J Gen Virol (2011) 92:2746–56. doi: 10.1099/vir.0.036004-0 21813708

[43] Bernard A. Lamy & Alberti, I. The two-signal model of T-cell activation after 30 years. Transplantation (2002) 73:S31–35. doi: 10.1097/00007890-200201151-00011 11810059

[44] Asashima H Mohanty S Comi M Ruff WE Hoehn KB Wong P. PD-1(high)CXCR5(-)CD4(+) peripheral helper T cells promote CXCR3(+) plasmablasts in human acute viral infection. Cell Rep (2023) 42:111895. doi: 10.1016/j.celrep.2022.111895 36596303 PMC9806868

[45] Cillo AR Somasundaram A Shan F Cardello C Workman CJ Kitsios GD. People critically ill with COVID-19 exhibit peripheral immune profiles predictive of mortality and reflective of SARS-CoV-2 lung viral burden. Cell Rep Med (2021) 2:100476. doi: 10.1016/j.xcrm.2021.100476 34873589 PMC8636386

