# Supplementary material for: Virus infection pattern imprinted and diversified the differentiation of T-cell memory in transcription and function
Source: Front Immunol. 2024 Jan 9;14:1334597. doi: 10.3389/fimmu.2023.1334597 (PMC10803622; doi:10.3389/fimmu.2023.1334597)
Supplement: Supplementary file 1 [file Image_1.pdf]

supplementary Figure 1

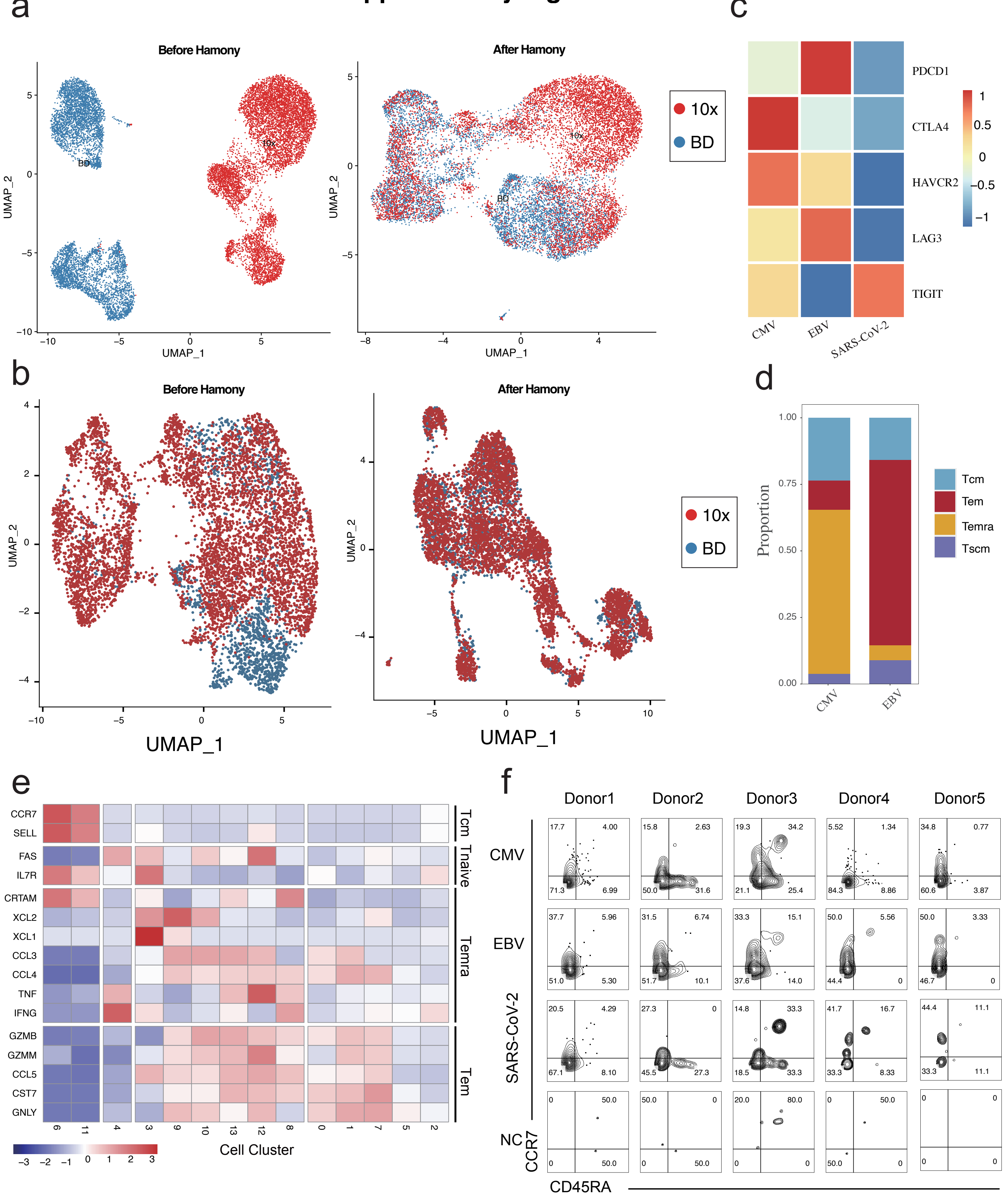

supplementary Figure 2

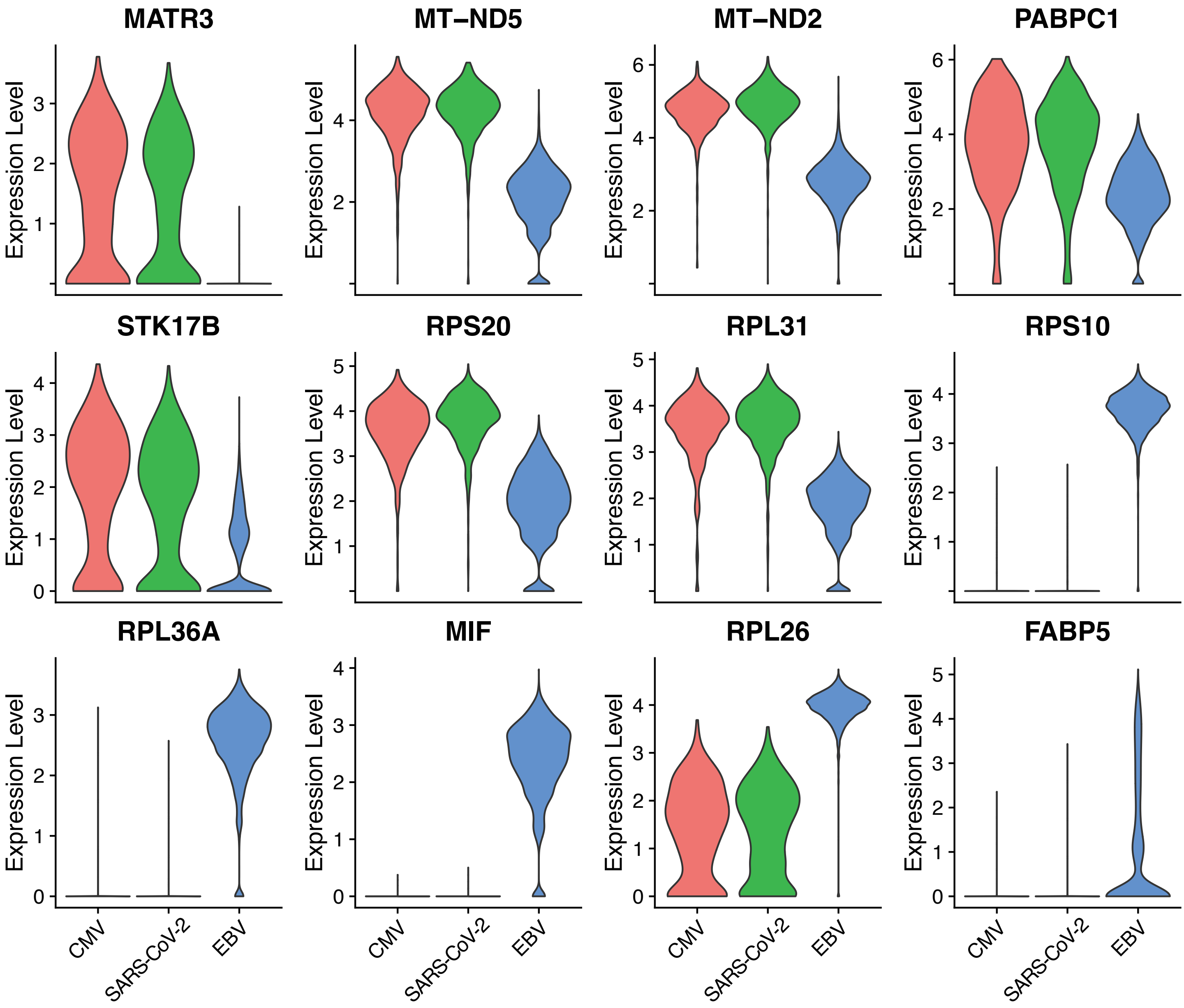

Supplementary Figure 3

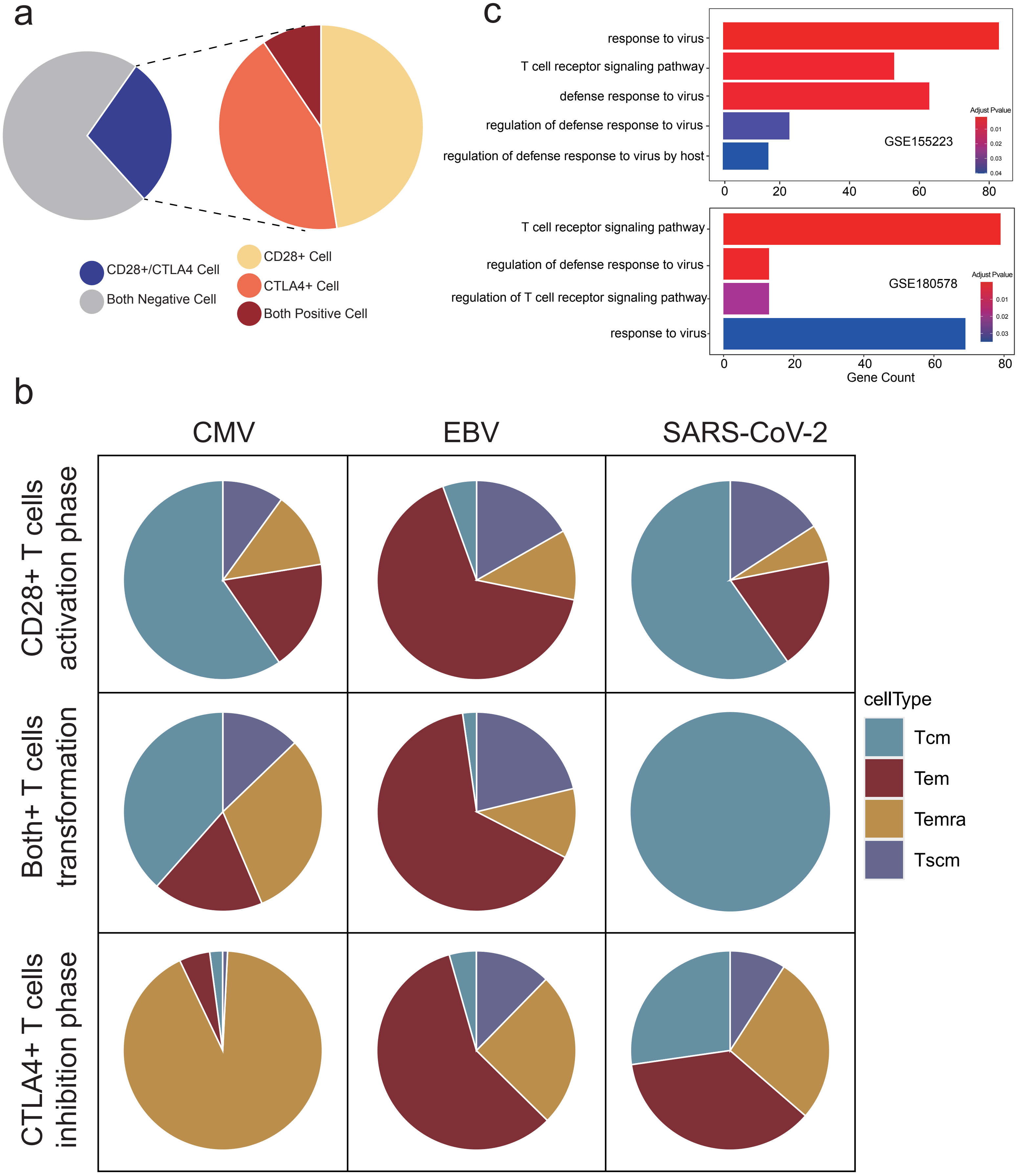

supplementary Figure 4

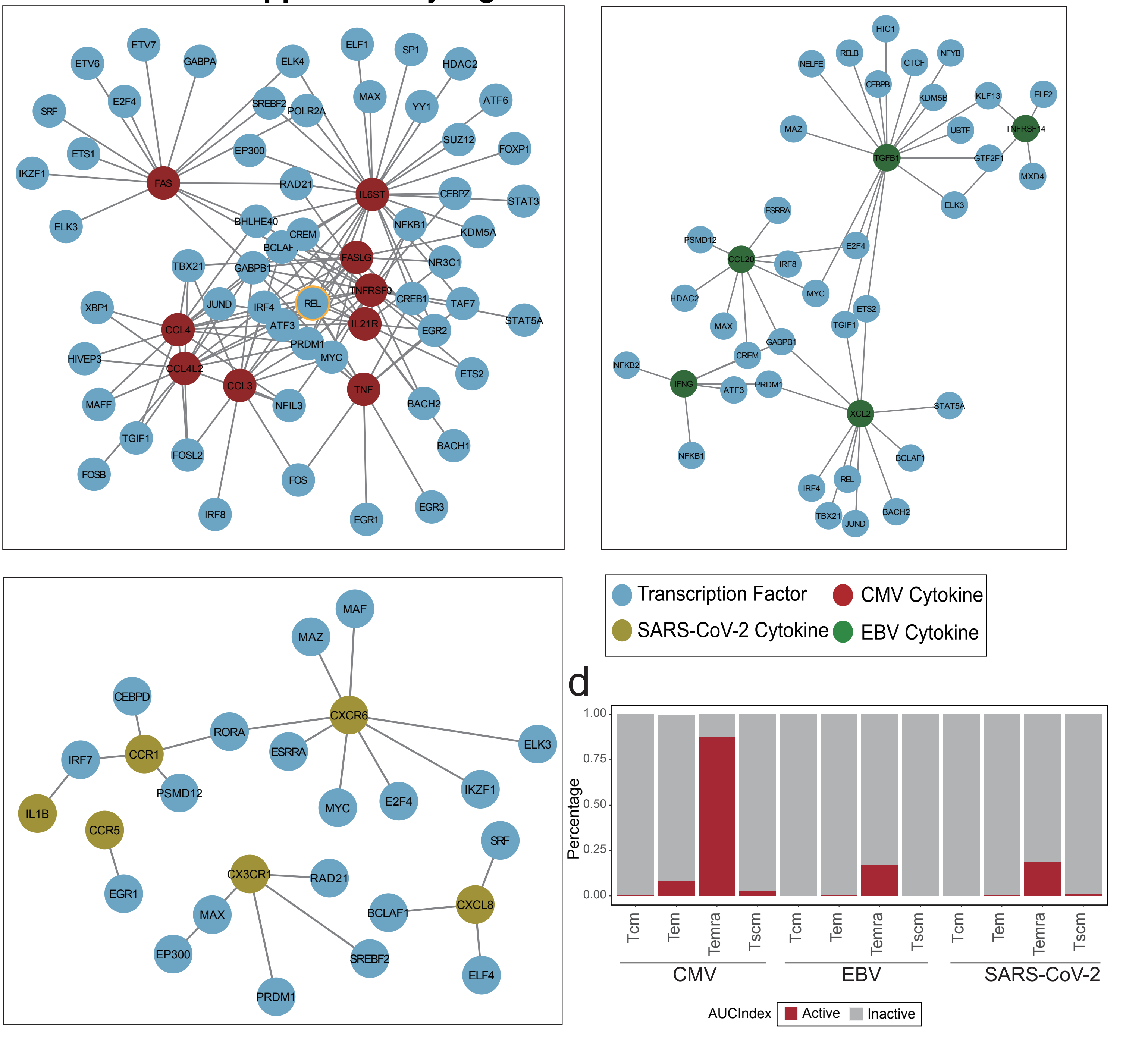

supplementary Figure 5

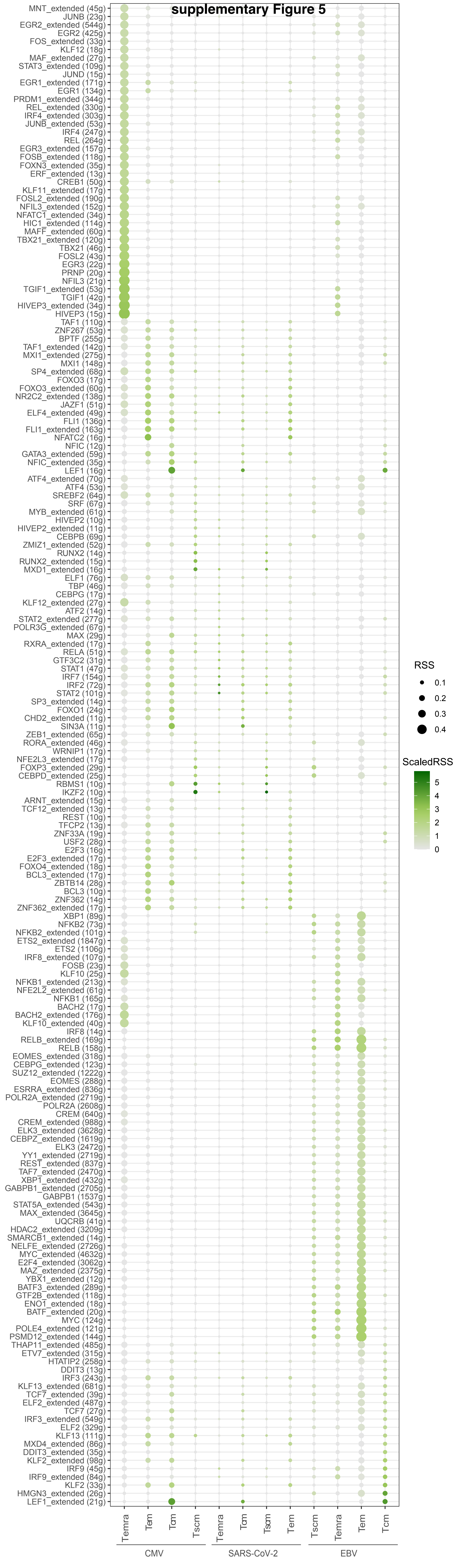

supplementary Figure 6

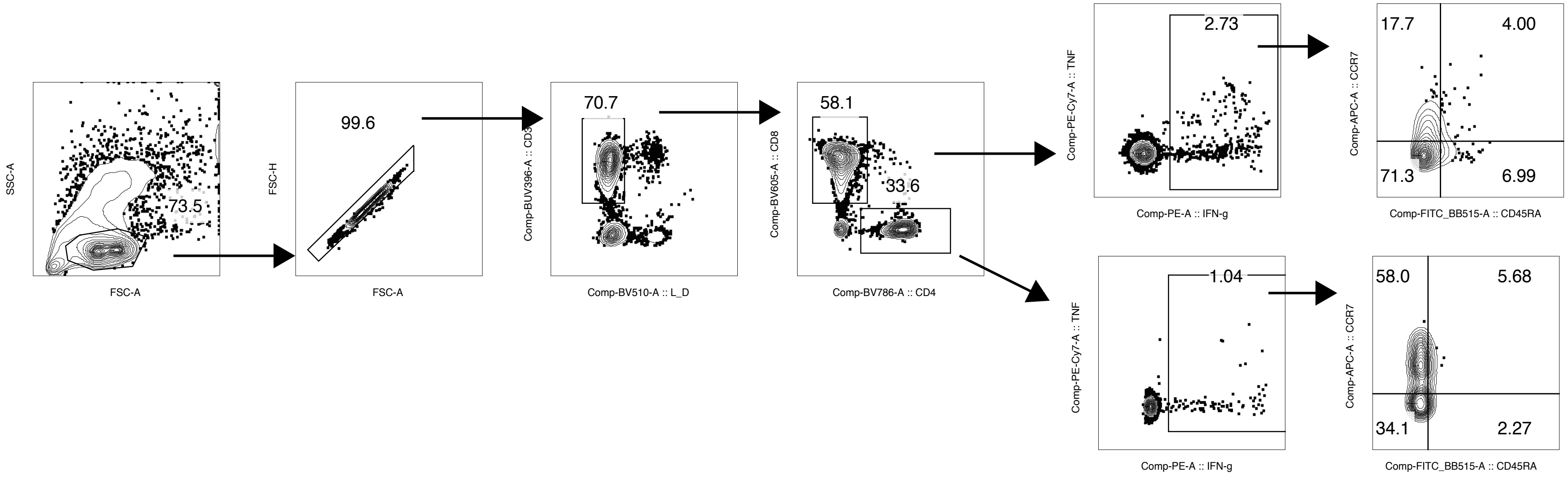

Supplementary Figure Legend

Supplementary Figure 1: The composition of T cell repertoires were different in CMV, EBV and SARS-CoV-2 in validation dataset. a) The UMAP of $CD8^+$ cells data from two different platform before 'Harmony' correction (left) and after 'Harmony' correction (right). b) silimar with that in figure a, but shows the data of $CD4^+$ cells. c) The heatmap shows the average gene expression for the genes listed in the right. The values were row scaled and represented by color shown in the legend. d) The proportion of different cell clusters in the T cell repertoire that primed with CMV pMHC and EBV pMHC in validation datasets. e) Clustering and cell type determination of memory T cells in validation dataset. Each single cells in the validation dataset were clustered into 0-13 clusters as listed under each column. The cell types were listed on the right of the heatmap and the marker genes used for cell type determination were listed on the left. The color for each square was assigned according to the mean expression of genes in all cells for each cluster. f) FACS plots showing the proportion of CD45RA+CCR7- (Temra) T cells and CD45RA-CCR7-(Tem) T cells in 5 convalescents in CMV-peptide, EBV-peptide and SARS-CoV-2 peptides stimulation models.

Supplementary Figure 2: Viral specific gene expression in CD4 T cells. The violin plots showed given gene expression distribution in virus-specific CD4 T cells. CMV CD4 T cells were plotted with red violin, EBV- were blue and SARS-CoV-2 were green.

Supplementary Figure 3: T cells with both CD28 and CTLA4 expression. a) The left pie plot shows the proportion of cell with either CD28 or CTLA4 expression. The right pie shows the proportion of cells expressing CD28, CTLA4 alone or both. b) Each single pie plot indicates the proportion of cell types in the T cell clusters with specified viral primed and specified cell state. c) The two barplots showed the enriched functions done with genes that positive correlated with ratio of CD28/CTLA4 in two independent datasets.

Supplementary Figure 4: TF-Cytokine network. a-c) The network constructed with virus specific cytokines in figure2.a and their SCENIC imputed transcription factors. Blue nodes are deduced TFs and the lines represent the regulation relationship between cytokine and TF. CMV specific cytokines were labeled with red dots in figure a. EBV specific cytokines were labeled with green dots in figure b. And SARS-CoV-2 were labeled with gold dots in figure c. d) According to the AUC cutoff labeled in figure 5.b, the cells were separated into REL regulon active (red) ones and inactive (gray) ones. Each barplot indicates the percentage of active and inactive cells in each virus-specific T cell clusters.

Supplementary Figure 5: Viral specific Regulons. All the SCENIC deduced TFs with their RSS scores in each virus-specific T cell subsets.

Supplementary Figure 6: Gating strategy for supplementary Figure1.f
